# Xuezhikang reduced arterial stiffness in patients with essential hypertension: a preliminary study

**DOI:** 10.1590/1414-431X20176363

**Published:** 2017-08-31

**Authors:** J. Zheng, T. Xiao, P. Ye, D. Miao, H. Wu

**Affiliations:** Department of Geriatric Cardiology, Chinese People's Liberation Army General Hospital, Beijing, China

**Keywords:** Arterial stiffness, Essential hypertension, Pulse wave velocity, Xuezhikang, Lipid profile

## Abstract

This study aimed to test the effects of xuezhikang, a cholestin extract that contains statin-like components, on arterial stiffness in patients with essential hypertension. One hundred hypertensive patients from the Chinese PLA General Hospital were randomly allocated to receive xuezhikang (1200 mg/day, orally) or placebo (same capsules containing only pharmaceutical excipients). Physical examination outcomes, lipid profile, high sensitivity C-reactive protein (hs-CRP) levels, matrix metalloproteinases-9 (MMP-9) levels, and arterial outcomes, including stiffness parameter (β), pressure-strain elasticity modulus (Ep), arterial compliance (AC), augmentation index (AI), and one-point pulse wave velocity (PWVβ) were obtained at baseline and after 6 months of the intervention. Xuezhikang significantly reduced β (8.4±3.1 *vs* 6.8±2.1, P=0.007), Ep (122.8±43.9 *vs* 100.7±33.2, P=0.009), PWVβ (6.7±1.2 *vs* 6.1±1.0, P=0.013), low-density lipoprotein cholesterol (3.4±0.6 *vs* 2.9±0.5, P=0.001), hs-CRP [2.1 (0.4-10.0) *vs* 1.4 (0.3-4.1), P=0.020], and MMP-9 (17.2±2.4 *vs* 12.7±3.8, P <0.001) compared to baseline. The placebo had no effect on these parameters. The changes of PWVβ in the xuezhikang group was significantly associated with the changes of hs-CRP and MMP-9 (r=0.144, P=0.043; r=0.278, P=0.030, respectively) but not with lipid profile changes. Our research showed xuezhikang can improve the parameters of arterial stiffness in hypertensive patients, and its effect was independent of lipid lowering.

## Introduction

Arterial stiffness, characterized by imbalances between vasodilator and vasoconstrictive factors, could result in increased pulse wave velocity (PWV), which is associated with functional sclerotic changes in elastic and collagen fibers of the arteries (1,2). Increased arterial stiffness is an early but non-necessary event in hypertension (2) and is considered to play a crucial role in cardiovascular events (3–6).

Statins are by far the most widely used lipid-lowering drugs (7). Compelling clinical trials and meta-analyses have shown primary and secondary prevention with statins, consistently showing reductions of cardiovascular events (7). However, the effects of statin therapy on PWV were not consistent in trials (8–10), with one study showing a significant increase in carotid-femoral PWV (cfPWV) after treatment (11).

Xuezhikang is a partial extract of red yeast rice, contains naturally occurring statins, and has good lipid modulating effect (12). Xuezhikang contains the 3-hydroxy-3-methylglutaryl coenzyme A (HMG-CoA) reductase inhibitor lovastatin, flavonoids, ergosterol amino acids, unsaturated fatty acids, trace elements, and other effective components. Xuezhikang efficiency was supported by the 2007 Guidelines on Prevention and Treatment of Blood Lipid Abnormality in Chinese Adults (GPTBLACA) based on the China Coronary Secondary Prevention study (13). Xuezhikang is an approved medication in China, mainly containing lovastatin, also termed monoclonin K, with 2.5-3.2 mg/capsule, a small amount of lovastatin hydroxyl acid, and ergosterol. It is a safe drug that can effectively lower the rate of coronary events, coronary death, and all-cause mortality.

This study aimed to explore the effects of xuezhikang on PWV in hypertensive patients.

## Patients and Methods

### Study population and design

The study was approved by the Ethics committee of the Chinese People's Liberation Army (PLA) General Hospital. Each participant provided a written informed consent. This prospective, randomized, single-blind, placebo-controlled study was performed in consecutive patients (n=100). Patients were enrolled at the Chinese PLA General Hospital from September 2010 to April 2011. Because the 2007 GPTBLACA and 2013 American College of Cardiology/American Heart Association guidelines state different criteria for the treatment of blood cholesterol between men and women, a different age criterion had to be used. The primary outcome in the current study was the arterial stiffness in subjects who received 6 months of xuezhikang treatment. The inclusion criteria were: 1) essential hypertension; 2) normal or mildly elevated serum lipid profile such as total cholesterol (TC) <6.19 mmol/L and/or low-density lipoprotein cholesterol (LDL-C) <4.12 mmol/L (14), and 3) aged ≥45 years for men or ≥55 years for women.

The exclusion criteria were: 1) secondary hypertension; 2) taking lipid-regulating agents such as statins, niacin, probucol, or fibrates; 3) coronary heart disease; 4) arrhythmia; 5) heart failure; 6) diabetes mellitus; 7) peripheral artery disease; 8) primary hyperthyroidism or hypothyroidism; 9) known intolerance or hypersensitivity to HMG-CoA reductase inhibitors; 10) alanine aminotransferase (ALT) or aspartate aminotransferase (AST) levels >2-fold the upper normal limit; 11) serum creatinine >178 µmol/L or creatine kinase (CK) >3-fold the upper normal limit; 12) Behcet disease, or 13) psoriasis.

### Sample consideration and intervention

In this study, sample sizes were calculated according to the possible lowered level of TC after patients were treated with placebo or Xuezhikang. The data were acquired in published research (15,16) and the PASS 11.0 software was used for sample size calculation. The calculated minimum sample size was 40 for each group.

The recruited patients first entered a 4-week run-in period during which they received a low salt and low fat diet, birth control, and moderate exercise. At the same time, patients were asked to refrain from the use of other blood lipid control agents such as fish oil or natto (a traditional Japanese food made from fermented soybeans). Subjects were allowed to take antiplatelet agents and antihypertensive drugs throughout the study period. All patients underwent the same treatment during this period. The patients were then randomly divided into two groups (n=50/group). Treatment allocation was prepared by a statistician using a random number table and sequential sealed envelopes. Patients received 1200 mg/day (600 mg in the morning, 600 mg in the evening, orally) of xuezhikang (WBL Peking University Biotech Co., Ltd., China) or a placebo (same capsules containing only pharmaceutical excipients). The drug or placebo allocation was blind to subjects. The treatment phase lasted 6 months. The patients were asked to maintain a stable diet in terms of types and amounts of nutrients according to the dietary advice they received. The patients continued all ongoing treatments such as aspirin, β-blockers, angiotensin converting enzyme inhibitors, and/or diuretics.

### Collection of clinical data

Demographic characteristics including lifestyle and medical history was collected. The medication history was obtained by self-reported standardized questionnaires. The investigation was completed by physicians who were trained by the research team. Anthropometric measurements were performed at the clinic, with participants wearing light clothing and no shoes. Height was measured in centimeters using a wall-mounted measuring tape. Weight was measured in kilograms using a digital scale. Systolic and diastolic blood pressures (BP) were measured by well-trained staff members on the right arm, in the sitting position, after at least 5 min of rest, with a mercury sphygmomanometer (Yuyue, Arma-mentarium Limited Company, China) and an appropriately sized cuff. Three sequential BP measurements were obtained, and the average was used for analyses. Body-mass index (BMI) was calculated as kg/m^2^. Hypertension was defined as a mean systolic BP ≥140 mmHg, and/or mean diastolic BP ≥90 mmHg, and/or use of antihypertensive medication (17).

### Laboratory measurements

After overnight fasting (>10 h), blood samples were collected in tubes containing separating gel for the measurement of the biomarkers. The samples were maintained at 4°C for <2 h before being centrifuged at 1200 *g* for 15 min at 4°C. Serum aliquots were frozen at -80°C. Levels of TC, LDL-C, triglycerides (TG), high-density lipoprotein cholesterol (HDL-C), ALT, AST, and CK were determined using Roche enzymatic assays (Roche Diagnostics GmbH, Germany) and a Roche auto-analyzer (Roche Diagnostics, USA). Levels of serum creatinine (Scr) were measured by an enzymatic assay (Roche Diagnostics GmbH) on a Hitachi 7600 auto-analyzer (Hitachi, Japan). Levels of high-sensitivity C-reactive protein (hs-CRP) was determined by an immunoturbidimetric assay (Siemens Healthcare Diagnostics, USA) using a Dimension RxL Max analyzer (Siemens Healthcare Diagnostics). Serum homocysteine (Hcy) levels were measured by a competitive immunoassay using direct chemiluminescence (Siemens Centaur Immunoassay Systems, USA). Serum matrix metalloproteinase-9 (MMP-9) levels were determined using a commercial ELISA kit (R&D Systems Inc., USA).

### Arterial stiffness

The parameters of arterial stiffness including stiffness parameter (β), pressure- strain elasticity modulus (Ep), arterial compliance (AC), augmentation index (AI), and one-point pulse wave velocity (PWVβ) were determined by an ultrasound echo-tracking system (Aloka α-10, Japan) with a 7.5-MHz linear array probe. Subjects were studied after 15 min of supine rest, in a temperature-controlled environment. Pressure waveforms were noninvasively obtained using arterial diameter change waveforms calibrated based on systolic and diastolic BP values measured with a cuff-type manometer applied to the upper arm.

Arterial stiffness parameters were automatically calculated as a mean of five heartbeats, according to established formulas (18):

β=In(Ps/Pd)/(Ds−Dd/Dd)

AC=π(Ds ×Ds−Dd×Dd)/4(Ps−Pd)

AI=[△P/(Ps−Pd)]×100

Ep=(Ps−Pd)/[(Ds−Dd)/Dd]

where Ps and Pd are systolic and diastolic BP values, Ds and Dd are the maximal and minimal diameters of the right common carotid artery, and ΔP is the difference between maximal BP and BP at the first peak (shoulder) on the carotid pressure waveform. PWVβ is derived from β (19).

### Statistical analyses

Categorical variables are reported as frequency and were analyzed using Fisher's exact test. Continuous variables are reported as means±SD. Comparisons between groups were performed with the independent sample Student's *t*-test or Wilcoxon/Mann-Whitney or chi-square test, when appropriate. Coefficients of correlation (r) were calculated by the Pearson correlation method. P<0.05 was considered to be statistically significant. All analyses were performed with SPSS 17.0 (IBM, USA).

## Results

### Characteristics of recruited patients

Five cases withdrew during the run-in period for refusing to provide blood samples. Five cases withdrew during treatment for failing to take the medicine on time or taking other statin drugs. A total of 90 patients completed the experiment successfully with 46 in the xuezhikang group and 44 in the placebo group. Baseline characteristics of these patients are reported in Table 1. Both groups were well-matched in regard to age, gender, current smoking, BMI, systolic and diastolic BP, lipid parameters, Scr, ALT, AST, and CK. There was no significant difference in the use of medications such as calcium channel blockers, β-blockers, angiotensin converting enzyme inhibitor, and diuretics at baseline between the two groups. Similarly, arterial stiffness parameters such as β, Ep, AC, AI, and PWVβ were comparable between the two groups at baseline.

**Table 1.** Baseline characteristics of one hundred hypertensive patients randomly allocated to receive xuezhikang (1200 mg/day) or placebo.

Variables	Xuezhikang (n=46)	Placebo (n=44)	P value
Age (years)	58.4±10.0	57.1±10.4	0.536
Males, years, (n)	57.9±9.7 (19)	56.7±10.1 (22)	0.701
Females, years, (n)	58.6±10.2 (27)	57.4±10.3 (22)	0.685
Male, n (%)	19 (41.3)	22 (50.0)	0.408
Current smoking, n (%)	11 (23.9)	12 (27.3)	0.715
BMI (kg/m2)	24.5±3.3	24.7±3.5	0.546
SBP (mmHg)	138.1±13.2	135.9±13.7	0.451
SBP in males	138.8±14.8	137.1±15.2	0.956
SBP in females	138.0±15.4	133.2±12.9	0.308
DBP (mmHg)	87.7±9.4	87.5±10.3	0.919
DBP in males	90.9±7.7	90.5±9.3	0.886
DBP in females	84.2±8.7	83.7±10.3	0.754
PP (mmHg)	50.5±13.3	48.5±12.3	0.476
TC (mmol/L)	5.5±0.7	5.3±0.8	0.194
TG (mmol/L)	2.6±1.6	2.4±1.4	0.539
LDL-C (mmol/L)	3.4±0.6	3.4±0.8	0.425
HDL-C (mmol/L)	1.2±0.2	1.2±0.3	0.852
Scr (µmol/L)	63.8±11.2	65.2±13.1	0.658
ALT (U/L)	21.2±6.4	22.5±8.8	0.467
AST (U/L)	26.2±13.8	26.5±14.2	0.923
CK (U/L)	108.0±67.5	107.3±48.8	0.961
Medication history, n (%)			
CCB use, n (%)	15 (32.6)	13 (29.5)	0.754
β-B use, n (%)	5 (10.9)	6 (13.6)	0.689
ACEI use, n (%)	6 (13.0)	6 (13.6)	0.934
Diuretic use, n (%)	1 (2.2)	2 (4.5)	0.612
Arterial stiffness parameters			
β	8.41±3.11	8.59±2.81	0.772
Ep	122.77±43.85	121.43±39.73	0.882
AC	0.74±0.24	0.80±0.31	0.271
AI	18.12±12.75	23.38±16.16	0.097
PWVβ	6.65±1.17	6.63±0.958	0.903

Data are reported as means±SD or number and percentages. BMI: body mass index; SBP: systolic blood pressure; DBP: diastolic blood pressure; PP: pulse pressure; TC: total plasma cholesterol; TG: triglyceride; LDL-C: low-density lipoprotein cholesterol; HDL-C: high-density lipoprotein cholesterol; Scr: serum creatinine; ALT: alanine aminotransferase; AST: aspartate aminotransferase; CK: creatine kinase; CCB: calcium channel blocker; β-B: β-blocker; ACEI: angiotensin converting enzyme inhibitor; β: stiffness parameter; Ep: pressure strain elasticity modulus; AC: arterial compliance; AI: augmentation index; PWVβ: one point pulse wave velocity. Statistical analyses were carried out with *t*-tests or chi-square test, when appropriate.

### Adverse reactions

No severe adverse reaction occurred during the treatment period. Mild abdominal distension occurred in 3 patients receiving xuezhikang. In one case, an elevated ALT occurred but not exceeding 2-fold the normal level (56 U/L, normal: 5-40 U/L). An elevated AST (67 U/L, normal: 0-40 U/L) occurred in 1 case receiving xuezhikang treatment.

### Changes of lipid levels, arterial stiffness parameters, and related variables after 6 months of treatment

In the xuezhikang group, the levels of TG, TC, and LDL-C were decreased significantly and HDL-C levels were increased significantly after 6 months of treatment. The artery stiffness parameters β, Ep, and PWVβ were decreased significantly after 6 months of xuezhikang compared to baseline, while AC and AI were not affected by xuezhikang. Compared with placebo treatment, xuezhikang significantly decreased β and Ep, while AC, AI and PWVβ in the 2 groups had no statistical difference after 6-months treatment. The levels of hs-CRP, Hcy, and MMP-9 were decreased significantly in response to xuezhikang (Supplementary Table S1).

The placebo did not significantly affect the lipid levels, except for TC. Furthermore, all arterial stiffness parameters and levels of hs-CRP, Hcy, and MMP-9 were not significantly changed after 6 months of placebo (all P>0.05; Supplementary Table S1).

### Correlation analyses

There was no significant correlation between the changes in arterial stiffness parameters and the changes in lipid parameters after treatment with xuezhikang (Table 2).

**Table 2.** Correlations between the changes of arterial stiffness parameters and lipid levels of 100 hypertensive patients randomly allocated to receive xuezhikang (1200 mg/day) or placebo.

	△TG (mmol/L)		△TC (mmol/L)		△LDL-C (mmol/L)		△HDL-C (mmol/L)	
	r	P value	r	P value	r	P value	r	P value
△β	0.099	0.420	0.011	0.926	0.055	0.657	-0.168	0.168
△Ep	0.050	0.690	0.016	0.901	0.031	0.810	-0.119	0.345
△AC	0.133	0.274	0.040	0.747	0.055	0.661	0.264	0.058
△AI	0.009	0.941	0.071	0.564	0.115	0.352	-0.030	0.806
△PWVβ	0.031	0.802	0.031	0.800	0.072	0.561	-0.125	0.308

TG: triglyceride; TC: total cholesterol; LDL-C: low-density lipoprotein cholesterol; HDL-C: high-density lipoprotein cholesterol; β: stiffness parameter; Ep: pressure: strain elasticity modulus; AC: arterial compliance; AI: augmentation index; PWVβ: one-point pulse wave velocity.

There were significant correlations between Δβ and Δhs-CRP, Δβ and ΔMMP-9, ΔEp and ΔMMP-9, ΔPWVβ and Δhs-CRP, and ΔPWVβ and ΔMMP-9 (Table 3).

**Table 3.** Correlations between the changes in arterial stiffness parameters and the changes in laboratory parameters of 100 hypertensive patients randomly allocated to receive xuezhikang (1200 mg/day) or placebo.

	△hs-CRP (mg/L)		△Hcy (µmol/L)		△MMP-9 (µg/L)	
	r	P	r	P	r	P
△β	0.125	0.039	0.064	0.571	0.319	0.044
△Ep	0.131	0.157	0.105	0.139	0.297	0.038
△AC	0.129	0.096	0.071	0.204	0.210	0.128
△AI	0.138	0.124	0.098	0.215	0.168	0.075
△PWVβ	0.144	0.045	0.177	0.157	0.278	0.030

hs-CRP: high-sensitivity C-reactive protein; Hcy: homocysteine; MMP-9: matrix metalloproteinase-9; β: stiffness parameter; Ep: pressure-strain elasticity modulus; AC: arterial compliance; AI: augmentation index; PWVβ: one-point pulse wave velocity.

## Discussion

In this study, xuezhikang significantly decreased the levels of TG, TC, and LDL-C, and increased HDL-C levels, which is consistent with previous studies (12,20). Furthermore, xuezhikang led to a significant reduction in arterial stiffness parameters such as β, Ep, and PWVβ and reduced the levels of hs-CRP, Hcy, and MMP-9. The changes of β, Ep, and PWVβ in the xuezhikang group were significantly correlated with the changes of hs-CRP or/and MMP-9, but not with the changes of the lipid profile. Although the comparison after treatment showed no statistical difference between the two groups in PWVβ, an arterial stiffness parameter, the P value was near significance (P=0.051) and it should be considered that the clinical samples were not calculated according to the data of arterial stiffness parameter. Our experiment may need to be verified in further study according to the current data. Our current research demonstrated that six months of xuezhikang treatment reduced the arterial stiffness parameters in patients with essential hypertension.

Interestingly, the changes of β, Ep and PWVβ in the xuezhikang group were significantly correlated with the changes of hs-CRP or MMP-9, but not with the changes of the lipid profile. Vascular stiffening occurs as a consequence of a complex interplay between several independent factors, as well as inter-dependent factors (21). The stiffening of the vessel has been associated with hemodynamic forces, the hormonal milieu, the intake of salt, and the individual's glycemic state etc. (22). It is assumed that statins exert their effects through cholesterol reduction (7,23). However, clinical trials have suggested that their benefits may extend beyond cholesterol, supporting the notion that a part of the clinical benefits of statins may be independent from LDL-C reduction (24–28). The JUPITER trial showed that statins could be beneficial in the primary prevention of cardiovascular disease in patients with elevated hs-CRP but with low cholesterol levels (23). Recent findings from the multicenter coronary atherosclerosis study measuring effects of rosuvastatin using intravascular ultrasound trial in Japanese subjects confirmed these data (29).

MMPs play an important role in remodeling the extra-cellular matrix. Increases of plasma MMP-9 are involved in the pathogenesis of early atherosclerosis and hypertension, and are probably one of the initiative factors of progressing arterial stiffness (30,31). This study showed that after 6 months of xuezhikang, levels of hs-CRP and MMP-9 decreased significantly and were significantly correlated with arterial stiffness parameters, indicating that xuezhikang may exert some anti-inflammatory and anti-vascular remodeling effects that are involved in improving arterial stiffness. Further trials will have to be performed to confirm these points.

In this study, the changes of arterial stiffness induced by xuezhikang did not correlate with the lipid-lowering effects. Since the subjects were hypertensive patients with normal or mildly increased blood lipid levels, the blood lipids were probably not affecting arterial stiffness at baseline. Indeed, high blood lipid levels increase arterial stiffness through the development of arterial plaque (32,33). In this study, ultrasound showed that only 12 (13.3%) patients had carotid arterial plaque, suggesting that the baseline effect of lipid levels on arterial stiffness was limited in this study.

This study is not without limitations. First, the sample size was limited and the observation period was only 6 months. Second, only ultrasound echo tracking was used to collect the arterial stiffness parameters. Although this method can overcome limitations of the traditional methods for arterial stiffness measurement such as cfPWV, no correlation analysis was performed between the two methods. Third, central aortic pressure had statistically significant relationships with serum calcium levels, phosphorus levels, magnesium levels, serum vitamin B12, and 24 h urinary microalbumin excretion rates in patients. Regrettably, these parameters were not measured in our study. Most of all, arterial stiffness parameters can be affected by atherosclerosis risk factors such as smoking, alcohol consumption, hypothyroidism, Behcet disease, psoriasis, diabetes and older age (34–37). Thus, further large-sample, strictly designed clinical research is still needed to verify this therapeutic effect.

In conclusion, our preliminary research showed that six months of xuezhikang treatment reduced arterial stiffness in patients with essential hypertension. This effect was independent from the lipid lowering effect of xuezhikang.

## Supplementary Material

Click here to view [pdf].
